# BMC Pediatrics reviewer acknowledgement 2015

**DOI:** 10.1186/s12887-016-0564-3

**Published:** 2016-02-13

**Authors:** Nawsheen Boodhun

**Affiliations:** BioMed Central, Floor 6, 236 Gray′s Inn Road, London, WC1X 8HB UK

## Abstract

The editors of *BMC Pediatrics* would like to thank all our reviewers who have contributed to the journal in Volume 15 (2015).

**Eivind Aadland**

Norway

**Hazreen Abdul Majid**

UK

**Yehia Abed**

Palestinian Territory

**Allistair Abraham**

USA

**Kabir Abubakar**

USA

**Jean Adams**

UK

**Olusegun Adebami**

Nigeria

**Samuel Adegoke**

Nigeria

**Rebecca Adkins**

USA

**Daniel Agardh**

Sweden

**Girdhar Agarwal**

India

**Daniel Aggio**

UK

**Carlo Agostoni**

Italy

**Azza Ahmed**

USA

**Kareem Airede**

Nigeria

**Sunday Ajike**

Nigeria

**Shamima Akhter**

Bangladesh

**Hasan Salih Zeki Aksu**

Turkey

**David Albayrak**

Turkey

**Ute Alexy**

Germany

**Ryan Allen**

Canada

**Stephen Allen**

UK

**Nicole Almenrader**

Italy

**Anthony Almudevar**

USA

**Jane Alsweiler**

New Zealand

**Sergio Amarri**

Italy

**Namasivayam Ambalavanan**

USA

**Lilliam Ambroggio**

USA

**Roland Ammann**

Switzerland

**Peter Anderson**

Australia

**Hak Lee Ang**

Malaysia

**Matina Angelopoulou**

USA

**Rachel Annunziato**

USA

**Vincenzo Antona**

Italy

**Jean-Luc Ardilouze**

Canada

**Hany Binti Mohd Ariffin**

Malaysia

**Donald M Arnold**

Canada

**Benjamin Arnold**

USA

**Stephen Aronoff**

USA

**Teresa Arrigo**

Italy

**Saadet Arsan**

Turkey

**Ehsan Aryan**

Iran

**Fayrouz Ashour**

USA

**Gershim Asiki**

Uganda

**Elizabeth Asztalos**

Canada

**Thomas Atkinson**

USA

**Massimo Attanasio**

USA

**Nicola Austin**

New Zealand

**Richard Auten**

USA

**Tommaso Aversa**

Italy

**Inge Axelsson**

Sweden

**Paluku Bahwere**

Malawi

**Dian Baker**

USA

**Deeksha Bali**

USA

**Akash Bang**

India

**Jinan Banna**

USA

**Jytte Banner**

Denmark

**Alan Barker**

UK

**Shari Barkin**

USA

**Jacqueline Barnes**

UK

**Benjamin Bar-Oz**

Israel

**Jonathan Barratt**

UK

**Louis Bartoshesky**

USA

**Mir Basir**

USA

**John Bass**

USA

**Renato Bassan**

Italy

**Laura Basterfield**

UK

**Anna Basu**

UK

**Rebecca Baum**

USA

**Lynn Bayne**

USA

**Alia Bazzy-Asaad**

USA

**David Bean**

Australia

**Brian Becknell**

USA

**Consuelo Beck-Sague**

USA

**Zijo Begic**

Bosnia and Herzegovina

**Philippe Bégin**

Canada

**Karen Benzies**

Canada

**Véronique Béréziat**

France

**Doris Bergen**

USA

**James Berkley**

UK

**Jon Bernstein**

USA

**Bhavneet Bharti**

India

**Shazia Bhat**

USA

**Ramesh Y Bhat**

India

**Rajendra Bhimma**

South Africa

**Ari Bitnun**

Canada

**Matthew Bizzarro**

USA

**Nilesh Bokil**

Australia

**Rebecca Bolen**

USA

**Roumiana Boneva**

USA

**Ernesto Bongarzone**

USA

**Francisco Bonilla**

USA

**Erika Borkoles**

Australia

**Marlene Borschel**

USA

**Arturo Borzutzky**

Chile

**Roseanna Bourke**

New Zealand

**James Boynton**

USA

**Peter Bradbeer**

New Zealand

**Mirko Brandes**

Germany

**Andras Bratincsak**

USA

**Joseph Braun**

USA

**Elizabeth Braunlin**

USA

**Vicky Breakey**

Canada

**Jean Brender**

USA

**Ekua Brenu**

Australia

**Sally Brinkman**

Australia

**Wendy Brodribb**

Australia

**Katherine Brown**

UK

**Susan Bruce**

USA

**Heather Brumberg**

USA

**Andreas Brunklaus**

UK

**Mariana Brussoni**

Canada

**Luc Bruyndonckx**

Belgium

**Ian Burgess**

UK

**Rachel Burke**

USA

**Alice Burnett**

Australia

**Monica Burnett**

USA

**Barbara Burns**

USA

**Matthew Bush**

USA

**William Buwembo**

Uganda

**Baltica Cabieses**

UK

**Christie Cabral**

UK

**Francesco Cadario**

Italy

**Jerrilyn Cambron**

USA

**Andrew Campbell**

USA

**Jessica Duncan Cance**

USA

**Stefan Cano**

UK

**Waldemar (Wally) Carlo**

USA

**Roxane Carr**

Canada

**Bernie Carter**

UK

**Marco Caruselli**

France

**Natalie Carvalho**

Australia

**Davide Carvalho**

Portugal

**Darla Castelli**

USA

**Raja Chakraborty**

India

**Martin C.W. Chan**

Hong Kong

**King Chung Derwin Chan**

UK

**Yee-Ming Chan**

USA

**Alvin Chang**

Singapore

**Karen Charlton**

South Africa

**Sanjay Chawla**

USA

**Gang Chen**

Australia

**Jiangang Chen**

USA

**Erika Cheng**

USA

**Ngai Cheung**

Australia

**Rosy Chhabra**

USA

**Andreas Chiabi**

Cameroon

**Aaron Chidekel**

USA

**Lucy Chimoyi**

South Africa

**Josephat Chinawa**

Nigeria

**Gaetano Chirico**

Italy

**Mohammod Chisti**

Bangladesh

**Steve Cho**

USA

**Judith E. Cho Lieu**

USA

**Jill Chorney**

Canada

**John Chuo**

USA

**Edward Ciaccio**

USA

**Julie Cichero**

Australia

**Stefan Ciurea**

USA

**Reese Clark**

USA

**Antonio Clavenna**

Italy

**Michelle Cleary**

USA

**Ronald Cohen**

USA

**Claire Coles**

USA

**Ronnie Thomas Collins Ii**

USA

**Allan Colver**

UK

**Alison Condliffe**

UK

**Fallon Cook**

Australia

**Suzette Cooke**

Canada

**David Cooke**

USA

**Kieran Cooley**

Canada

**Roberto Copetti**

Italy

**Seth J Corey**

USA

**Barbara Cormack**

New Zealand

**Eva Corpeleijn**

Netherlands

**Dina Cortes**

Denmark

**Luciane Costa**

Brazil

**Sherry Courtney**

USA

**Tina Cowan**

USA

**Georgina Cox**

Australia

**Dianne Creighton**

Canada

**Alexandre Cristino**

Australia

**Solveig Cunningham**

USA

**Aubrey Cunnington**

UK

**Nagib Dahdah**

Canada

**Susan V. Dahinten**

Canada

**Frederic Dallaire**

Canada

**Annet Dallmeijer**

Netherlands

**Amy Damashek**

USA

**Anne-Sophie Darlington**

UK

**Rita D'Ascenzo**

Italy

**Reza Daugherty**

USA

**Enza D'Auria**

Italy

**Zoe Davidson**

Australia

**Sean Davies**

USA

**Fernando Maria De Benedictis**

Italy

**Anthony De Buys Roessingh**

Switzerland

**Gaston De Serres**

Canada

**Gabriele De Vos**

USA

**Tamas Decsi**

Hungary

**Iyabode Olabisi Dedeke**

Nigeria

**Andrew Degnan**

USA

**Walter Dehority**

USA

**Johnny Deladoey**

Canada

**Marie Delnord**

France

**Johan Delport**

Canada

**Eugene Dempsey**

Ireland

**Craig Derkay**

USA

**Shalini Desai**

Canada

**Chris Desmond**

South Africa

**Sangappa Dhaded**

India

**Mamadou Otto Diallo**

USA

**Victoria Dimitriades**

USA

**Ginette Dionne**

Canada

**Philippe Dixon**

USA

**Jennifer Doering**

USA

**Umit Bilge Dogan**

Turkey

**Siobhan Dolan**

USA

**Shaul Dollberg**

Israel

**Samuel Dominguez**

USA

**Caroline Donovan**

Australia

**Jon Dorling**

UK

**Sinisa Dovat**

USA

**Therese Dowswell**

UK

**Olivier Drouin**

USA

**Jennifer Duchon**

USA

**Akilah Dulin-Keita**

USA

**Carlos Duran**

Vanuatu

**Jimmy Efird**

USA

**Kamal Eldeirawi**

USA

**Reda Elgazzar**

Canada

**Ann-Christin Eliasson**

Sweden

**Jerome Elusiyan**

Nigeria

**Iqbal Elyazar**

Indonesia

**Clifton Emery**

South Korea

**Cyril Engmann**

USA

**Karin Enskär**

Sweden

**Elizabeth Epstein**

USA

**Susanna Esposito**

Italy

**Marg Ewen**

Netherlands

**Pam Factor-Litvak**

USA

**Yu Fang**

China

**Evaggelia Fappa**

Greece

**Adekunbi Farotimi**

Nigeria

**Edward Vincent Faustino**

USA

**Kenneth Feldman**

USA

**Ama Fenny**

Ghana

**Linda Fetters**

USA

**Martijn Finken**

Netherlands

**Federico Fiori**

UK

**Saker Firas**

USA

**Gadi Fishman**

Israel

**Sue Fletcher-Watson**

UK

**Adrienne Foran**

Ireland

**René Fortelny**

Austria

**Gary Foster**

Canada

**Nathan Fountain**

USA

**Sotirios Fouzas**

Greece

**Gustavo Fraga**

Brazil

**Richard Oscar Francis**

USA

**Robert Franco**

USA

**Melinda Franettovich Smith**

Australia

**Richard Franklin**

Australia

**Bonita Franklin**

USA

**Angela Fraser**

USA

**Michael Freeman**

USA

**Robert D. Friedberg**

USA

**Jens Fuglsang**

Denmark

**Takao Fujisawa**

Japan

**Jun Fujishiro**

Japan

**Jens Gaab**

Switzerland

**Dara Galagher**

Ireland

**Dario Galante**

Italy

**Chandika Damesh Gamage**

Japan

**Chandika Gamage**

Japan

**Javier Ganame**

Canada

**Livia Garavelli**

Italy

**Sandra Garcia**

Colombia

**Yvonne Garza**

USA

**Paul Wenzel Geissler**

Norway

**Christine Marie George**

USA

**Mohamed Ghorbel**

UK

**Maria Gianni'**

Italy

**Andrew Gill**

Australia

**Simone Gill**

USA

**Wendy Gilleard**

Australia

**Cathleen Gillespie**

USA

**Tabither Gitau**

Kenya

**Camilla Gizzi**

Italy

**Melissa Gladstone**

UK

**Jean-Philippe Godin**

Switzerland

**June Anne Gold**

Canada

**Karina Gomes**

Brazil

**Antonia Gómez Conesa**

Spain

**Linda Granowetter**

USA

**Eva Greibe**

Denmark

**Ian Griffin**

USA

**Oswin Grollmuss**

France

**Michelle Jennifer Groome**

South Africa

**Sareen Gropper**

USA

**Ornella Guardamagna**

Italy

**Anna Gudmundsdottir**

Sweden

**Ursula Guillen**

USA

**Mintaze Gunel**

Turkey

**Ingibjorg Gunnarsdottir**

Iceland

**Shuchita Gupta**

India

**Munish Gupta**

USA

**Shailvi Gupta**

USA

**Elena Gutierrez-Farewik**

Sweden

**Gerda-Maria Haas**

Germany

**Jean-Pierre Habicht**

USA

**Jess Haines**

Canada

**Wendy Hall**

Canada

**Patricia Hall**

USA

**Laura Hammitt**

USA

**James Hansen**

USA

**Regina Harbourne**

USA

**Deirdre Harrington**

UK

**Denise Harrison**

Canada

**Mohammad Hasan**

Qatar

**Janet Hauck**

USA

**Lize Havemann-Nel**

South Africa

**Steven Hawken**

Canada

**Nicola Hawley**

USA

**Shogo Hayashi**

Japan

**Alison Hayes**

Australia

**Tom Hazell**

Canada

**Jill Heathcock**

USA

**Mark Hector**

UK

**Ann Hermansson**

Sweden

**Mary Jo Cooley Hidecker**

USA

**Takahiro Higashi**

Japan

**Amanda Highet**

Australia

**Blanca Himes**

USA

**Erica Hinckson**

New Zealand

**Claas Heinrich Hinze**

USA

**Ravi Hiremagalore**

India

**Gerrit Hirschfeld**

Germany

**Adam Hittelman**

USA

**Michel Hof**

Netherlands

**Daniel Hoffman**

USA

**Rebecca Hommer**

USA

**Julie Hoover-Fong**

USA

**Rosemary Horne**

Australia

**Kembra Howdeshell**

USA

**Tasha Howe**

USA

**Erin Howie**

Australia

**Terry Huang**

USA

**Balthasar Hug**

Switzerland

**Jeffery Hughes**

Australia

**Denise Hughes**

USA

**Debbie Humphries**

USA

**Mohamed Hussein**

Egypt

**Brendon Hyndman**

Australia

**Silvia Iacobelli**

France

**Bede Ibe**

Nigeria

**Scott Ickes**

USA

**María Esther Irigoyen**

Mexico

**Iman Iskander**

Egypt

**Marina Ito**

Brazil

**Lorenzo Iughetti**

Italy

**Lindsay Jaacks**

USA

**Ajay Jain**

USA

**Sandeep G. Jakhere**

India

**Muhammad Yazid Jalaludin**

Malaysia

**Andrew James**

Canada

**Marilyn James**

UK

**Hamid Jan B. Jan Mohamed**

Malaysia

**Aron Janssen**

USA

**Michael Jantz**

USA

**Ashley Jayapaul**

Singapore

**Channa Jayasena**

UK

**Yuan Jiang**

USA

**Zeng Jianping**

China

**Elizabeth Jimenez**

USA

**Dipesalema Joel**

Botswana

**Liane Johnson**

Canada

**Lamis Jomaa**

Lebanon

**Christine Jones**

UK

**Jill Kaar**

USA

**Fatima Kakkar**

Canada

**Satish Kalhan**

USA

**Natarajaseenivasan Kalimuthusamy**

India

**Pradip Kamat**

USA

**Jane Kamau**

Kenya

**Yogavijayan Kandasamy**

Australia

**Edmund Wedam Kanmiki**

Ghana

**Kamila Kantovitz**

Brazil

**Vishal Kapadia**

USA

**Warren Kaplan**

Switzerland

**Paul Kaplowitz**

USA

**Bryan Karazsia**

USA

**Walter Karlen**

Switzerland

**Anup Katheria**

USA

**Noriko Kato**

Japan

**David Kaufman**

USA

**Lauren Kearney**

Australia

**Erin Kelly**

Australia

**Han Cg Kemper**

Netherlands

**Robert Kennedy**

USA

**Marko Kerac**

UK

**Claire Kerr**

Australia

**Sarah Kertz**

USA

**Norman Kettner**

USA

**Robinder Khemani**

USA

**Elizabeth Kibaru**

Kenya

**Katarzyna Kilis-Pstrusinska**

Poland

**Seung Kim**

Democratic People's Republic of Korea

**Kwang-Youn Kim**

USA

**Dimitra Kiritsi**

Germany

**Motomitsu Kitaoka**

Japan

**Sabine Klaassen**

Germany

**Mariska Klein Velderman**

Netherlands

**Rachel Knowles**

UK

**Anastasios Kollias**

Greece

**Ourania Kolokotroni**

Cyprus

**Bart Koot**

Netherlands

**David Korones**

USA

**Susanne Kost**

USA

**Nancy Krebs**

USA

**Vicki Kristman**

Canada

**Janet Krska**

UK

**Herculina Salome Kruger**

South Africa

**Elena Krumova**

Germany

**Madhuri Kulkarni**

India

**Surinder Kumar**

India

**Rashmi Kumar**

India

**Seema Kumar**

USA

**Diederik Kuster**

Netherlands

**Kenya Kusunose**

Japan

**Peter Kuti**

Nigeria

**Matthew Kwan**

Canada

**Veerle Labarque**

Belgium

**Harrie Lafeber**

Netherlands

**Yvonne Lamers**

Canada

**Claudio F. Lanata**

Peru

**Thorsten Langer**

Germany

**Paul Lantos**

USA

**Brittany Lapin**

USA

**Abbot Laptook**

USA

**Irene Lara-Corrales**

Canada

**Emma Larkin**

USA

**Richard Larouche**

Canada

**Charles Larson**

Canada

**Matthew Laughon**

USA

**Joshua Lawson**

Canada

**Avula Laxmaiah**

India

**Kirsty Le Doare**

South Africa

**Nicole Le Saux**

Canada

**Julia Lechuga**

USA

**Henry Lee**

USA

**Kyung-Yil Lee**

South Korea

**Sue Lee**

Thailand

**Kai Lehmberg**

Germany

**Venla Lehti**

Finland

**Bertrand Lell**

Germany

**Lucy Lemare**

Canada

**Brigitte Lemyre**

Canada

**Johannes Leonhardt**

Germany

**Scott Levin**

USA

**Ho Cheung William Li**

China

**Richard Lichenstein**

USA

**Karen Liller**

UK

**Ziyue Liu**

USA

**Rakesh Lodha**

India

**Nicola Lowe**

UK

**Lesley Lowes**

UK

**Linda Lowes**

USA

**Adam Lowry**

USA

**Laura Lucaccioni**

Italy

**Jonas Ludvigsson**

Sweden

**Bryan Luikart**

USA

**Joachim Luthander**

Sweden

**Amy Lynch**

USA

**Noni Macdonald**

Canada

**Heather Macdonald**

Canada

**Judith Macdonnell**

Canada

**Joanna Maclean**

Canada

**Rabiya Majeed**

UK

**Denise Manica**

Brazil

**Eirini Manthou**

Greece

**Louise Maranda**

USA

**Ilaria Mariotti**

Italy

**Alda Marques**

Portugal

**Marie Marshall**

UK

**Mary Martinasek**

USA

**Pierluigi Marzuillo**

Italy

**Wilbert Mason**

USA

**Tyler Mason**

USA

**Michael Mathai**

Australia

**Mogomotsi Matshaba**

Botswana

**Alicia Matthews**

USA

**Mumtaz M Mazicioglu**

Turkey

**Silvia Mazzoni**

Italy

**Jennifer Mcarthur**

USA

**David H. Mccarthy**

UK

**Hugh Mccarthy**

UK

**Eric Mccollum**

USA

**David Mccormick**

USA

**Heather Mcdermid**

Canada

**Jane Mcgowan**

USA

**Kenneth Mcintosh**

USA

**Audrey Mckinlay**

New Zealand

**Thomas Mclean**

USA

**Beth Mcmanus**

USA

**Stephen Edward Mcmillin**

USA

**Catherine Mcneal**

USA

**Amy Mcpherson**

Canada

**Cristina Mei**

Australia

**Karen Meir**

Israel

**Marian Melish**

USA

**Judith Mercer**

USA

**Kim Meredith-Jones**

New Zealand

**Darcey Merritt**

USA

**Patrick M. Meyer Sauteur**

Switzerland

**Blaise Meyrat**

Switzerland

**David Michalik**

USA

**Janet Mifsud**

UK

**Čižman Milan**

UK

**Gregorio Milani**

Italy

**Matthieu Million**

France

**David Mills**

USA

**Madhukar Mittal**

USA

**Maurice B Mittelmark**

Norway

**Phoenix Mo**

Hong Kong

**Angelika Mohn**

Italy

**Olugbenga Mokuolu**

Nigeria

**Sigvard Mölstad**

Sweden

**Adriana Montaño**

USA

**Makama Andries Monyeki**

South Africa

**Anita Morandi**

Italy

**Carmen Moreno**

Spain

**Ichiro Morioka**

Japan

**Michael Moritz**

USA

**Raffaella Mormile**

Italy

**Shaun Morris**

Canada

**Kevin Mortimer**

UK

**Cynthia Morton**

USA

**Moti Moskovitz**

Israel

**Cheryle Moss**

Australia

**Anja Mowes**

USA

**Michael Msall**

USA

**Tingwei Mu**

USA

**Paul Mullan**

USA

**Gillian Mulvale**

Canada

**David Murdoch**

New Zealand

**Stella Muthuri**

Canada

**Jayasree Nair**

USA

**Nader Nassif**

Italy

**Gunnar Naulaers**

Belgium

**Theo Nell**

South Africa

**Georges Nemer**

Lebanon

**Michael Nemergut**

USA

**Steve Nesheim**

USA

**Madalynn Neu**

USA

**Karen New**

USA

**Yvonne Peng Mei Ng**

Singapore

**Honor Nicholl**

Ireland

**Stefan Nilsson**

Sweden

**Fidelis Njokanma**

Nigeria

**Peter Nkwo**

Nigeria

**Jane Noyes**

UK

**Paolo Nucci**

Italy

**Altacilio Nunes**

Brazil

**Sivakumar Nuvvula**

India

**Bright Nwaru**

UK

**Chinomso Nwozichi**

Nigeria

**John O' Toole**

Ireland

**Ijeoma Obum-Anyim**

Nigeria

**Sophie Ochola**

Kenya

**Amanda Ogilvy-Stuart**

UK

**Rohit Ojha**

USA

**Angela Okolo**

Nigeria

**James Oleske**

USA

**Elisabeth Olhager**

Sweden

**Olufemi Oloyede**

Nigeria

**Jacob Olson**

USA

**Bolajoko O. Olusanya**

Nigeria

**Patricia O'Malley**

USA

**Michael O'Neill**

Ireland

**Jennifer O'Neill**

USA

**Dickens Onyango**

Kenya

**Antony Simon Opwora**

Kenya

**Yaw Osafo**

Ghana

**Irene Osborn**

USA

**Damjan Osredkar**

Norway

**Seth Owusu-Agyei**

Ghana

**Jennifer Oxley**

Australia

**Denise Page**

Australia

**Fauzia Paize**

UK

**Zenggang Pan**

USA

**Anand Pandey**

India

**Eleni Papadopoulou**

Norway

**Prabhu Parimi**

USA

**Samantha Parker**

USA

**Ravi Patel**

USA

**Viviana Patianna**

Italy

**Jaishree Paul**

India

**Dino Pedrotti**

Italy

**Lorine Pelly**

Canada

**Sihua Peng**

China

**Ji-Bin Peng**

USA

**Luis Pereira-Da-Silva**

Portugal

**Jeffrey Pernica**

Canada

**Michael Pichichero**

USA

**Cynthia Pileggi-Castro**

Brazil

**Rajamohanan Pillai**

India

**Richard Pinder**

UK

**Orit Pinhas-Hamiel**

Israel

**Joaquim Pinheiro**

USA

**Daniele Piovani**

Italy

**Jodie Plumert**

USA

**Patricia Pohl**

USA

**Alexey Polonikov**

Russian Federation

**Jon Polonsky**

France

**Emma Pomeroy**

UK

**Michael Portman**

USA

**Michael Posencheg**

USA

**Julie Postma**

USA

**Alex Postma**

Netherlands

**Elizabeth Powell**

USA

**Akila Prashant**

India

**Andrew Prendergast**

UK

**Amanda Price**

USA

**Lynne Prince**

UK

**Flavia Prodam**

Italy

**Evan Propst**

Canada

**Giuseppe Puccio**

Italy

**Shamim Qazi**

Switzerland

**Jose Bernardo Quintos**

USA

**Lesliam Quiros-Alcala**

USA

**Yacov Rabi**

Canada

**Mohammad Rafi**

USA

**Francesco Raimondi**

Italy

**Aparna Ramasubramanian**

USA

**Sharon Ramey**

USA

**Meenal Rana**

USA

**Saritha Ranabothu**

USA

**Janis Randall Simpson**

Canada

**Reeta Rasaily**

India

**Jennifer Reath**

Australia

**Catherine Rechnitzer**

Denmark

**Clare Rees**

Australia

**Christopher Alan Reid**

Australia

**Marvin Reid**

Jamaica

**Aiguo Ren**

China

**Raffaele Renella**

Switzerland

**Amy Renwick**

USA

**Daiane Resende**

Brazil

**Brian Rice**

USA

**Field Rickards**

Australia

**Nicola Ridgers**

Australia

**Laura Rindi**

Italy

**Tamar Ringel-Kulka**

USA

**Ashley Roberts**

Canada

**Jeff Robison**

USA

**Christine Rodda**

Australia

**Rosa Rodriguez-Monguio**

USA

**Paola Roggero**

Italy

**Cristiano Rosafio**

Italy

**Yale Rosen**

USA

**Ana Ruiz-Casado**

Spain

**Gianni Russo**

Italy

**Khaled Saad**

Egypt

**Mohammad Reza Sabri**

Iran

**Manish Sadarangani**

Canada

**Sandeep Sadashiv**

USA

**Ramesh Sahani**

India

**Reza Saidi**

USA

**Tolulope Sajobi**

Canada

**Rie Sakai**

USA

**Mio Sakuma**

Japan

**Ariel Salas**

Bolivia

**Irving Salit**

Canada

**Judith Sanchez-Manubens**

Spain

**Guilherme Sant'Anna**

Canada

**Mulkey Sarah**

USA

**Benn Sartorius**

South Africa

**Thomas Sato**

USA

**Laura Sauve**

Canada

**Teresa Savage**

USA

**Dennis Savaiano**

USA

**Hendrik Simon Schaaf**

South Africa

**Federico Schena**

Italy

**Lindsay Schenkel**

USA

**Angela Scheuerle**

USA

**Petra Schnell-Inderst**

Austria

**Pamela Schuetze**

USA

**Jane Scott**

Australia

**Michael Seckeler**

USA

**Janet Serle**

USA

**Guogen Shan**

USA

**Seetha Shankaran**

USA

**Chevis Shannon**

USA

**Deepak Sharan**

India

**Iman Sharif**

USA

**Michael Sherman**

USA

**Ajoy Prasad Shetty**

India

**Joseph Shieh**

USA

**Masaki Shimizu**

Japan

**Chisato Shimizu**

USA

**Sandesh Shivananda**

Canada

**Helen Shoemark**

Australia

**Michelle Short**

Australia

**Robert Sidbury**

USA

**Kamran Siddiqi**

UK

**Peter Sidebotham**

UK

**Ivani Silva**

Brazil

**Vinaya Simha**

USA

**Barbara Siminovich-Blok**

USA

**Victoria Simms**

UK

**Kari Simonsen**

USA

**Judy Simpson**

Australia

**Richard Sindelar**

Sweden

**Rahul Raman Sing**

UK

**Yogen Singh**

UK

**Chutima Sirikulchayanonta**

Thailand

**Seter Siziya**

Zambia

**Joseph Skelton**

USA

**Sheri-Lynn Skwarchuk**

Canada

**Jonathan Slaughter**

USA

**Deborah L. Slawson**

USA

**Nancy L Sloan**

USA

**Hans-Christian Slotved**

Denmark

**Tina Slusher**

Nigeria

**Mary Lou Smith**

Canada

**Drew Smith**

USA

**Sandra Smith**

USA

**Howard Snyder**

USA

**Samara Soghoian**

Ghana

**Sajid Soofi**

Pakistan

**Bridget Southwell**

Australia

**Ulla Sovio**

UK

**Takashi Sozu**

Japan

**Michael Spear**

USA

**Paul Spearman**

USA

**Carsten Speckmann**

Germany

**Angelica Spotti**

Italy

**Ben Spycher**

Switzerland

**Jane Squires**

USA

**Vijay Srinivasan**

USA

**Rosarin Sruamsiri**

Thailand

**Amanda Staiano**

USA

**Jamie Stang**

USA

**Ric Steele**

USA

**Philippe Steenhout**

Switzerland

**Pernilla Stenström**

Sweden

**Christine Stewart**

USA

**David Stodden**

USA

**Elisabeth Stoltz Sjostrom**

Sweden

**Jana Strahler**

Germany

**Lahn Straney**

Australia

**Robyn Stremler**

Canada

**Amelie Stritzke**

Canada

**Konstantinos Stroumpoulis**

Greece

**Kevin Sullivan**

USA

**Susan Sullivan-Bolyai**

USA

**Guibo Sun**

Hong Kong

**Jonathan R. Swanson**

USA

**Fern Tablin**

USA

**Yaser Taghipour Bazargani**

Netherlands

**Mohamed Tagin**

Canada

**Taro Takeshima**

Japan

**Tim Takken**

Netherlands

**Kenneth Tan**

Australia

**Behailu Tariku**

Ethiopia

**Mary Tavares**

USA

**Gabriela Tavchioska**

Macedonia

**Catherine Taylor**

Australia

**Richard Taylor**

Canada

**Sarah Taylor**

USA

**Amy Taylor**

UK

**Eva Thomas**

Canada

**David Richard Thomas**

Qatar

**Marie Thomas**

UK

**Vincent Thomas De Montpreville**

France

**John Michael David Thompson**

New Zealand

**Greg Tiao**

USA

**Andrew Tomkins**

UK

**Connie Tompkins**

USA

**Olukemi Tongo**

Nigeria

**Mahmoud Torabi**

Canada

**Susan B Torrey**

USA

**Vasiliki Totsika**

UK

**Jillian Trabulsi**

USA

**Leonardo Trasande**

USA

**Daniele Trevisanuto**

Italy

**Allan Truant**

USA

**Ilse Truter**

South Africa

**Elena Tsourdi**

Germany

**Kirsten Tulchin-Francis**

USA

**Robert Tulloh**

UK

**Paul Turner**

Cambodia

**Stuart Turvey**

Canada

**Deborah Tuttle**

USA

**Marina Ulanova**

Canada

**Mark Underwood**

USA

**Jean-Philippe Vaccani**

Canada

**Varun Vaidya**

USA

**Giuliana Valerio**

Italy

**Guy Van Vliet**

Canada

**Jillon Vander Wal**

USA

**Amber Vaughn**

USA

**Sukumar Vellakkal**

India

**Raveenthiran Venkatachalam**

India

**Maximo Vento**

Spain

**Stefania Vergnano**

UK

**Amber Vermeesch**

USA

**Ruud Vermeulen**

Netherlands

**Gestur Vidarsson**

Netherlands

**Susanne Vijverberg**

Netherlands

**Maryann Volpe**

USA

**Carl Von Baeyer**

Canada

**Dalton Wamalwa**

Kenya

**Gustavo Wandalsen**

Brazil

**Nancy Wang**

USA

**Dianne Ward**

USA

**Klaas Wardenaar**

Netherlands

**David Warhurst**

UK

**Susan Waserman**

Canada

**Malgorzata Wasniewska**

Italy

**Toru Watanabe**

Japan

**Erin Way**

USA

**Ashley Weaver**

USA

**Dianne Webster**

New Zealand

**Arie Weinstock**

USA

**Constance G Weismann**

USA

**Scott Weiss**

USA

**Martha Welch**

USA

**Yeo Tsin Wen**

Singapore

**Kurt Widhalm**

Austria

**Susan Wiegers**

USA

**Linda Wijlaars**

UK

**Kyle Wilby**

Qatar

**Duncan Wilcox**

USA

**Barbara Wildhaber**

Switzerland

**Veronica Wiley**

Australia

**Mark Willems**

UK

**Christopher Williams**

Australia

**Ruth Williams**

UK

**Benjamin Willing**

Canada

**Esko Wiltshire**

New Zealand

**Stephen Wong**

Hong Kong

**Charlotte Wright**

UK

**Clyde Wright**

USA

**Jiunn-Liang Wu**

Taiwan

**Rebecca Wyse**

Australia

**Ramesh Y Bhatt**

India

**Kapil Yadav**

India

**Kala Yadhav**

India

**Venkat Laxmi Yeruva**

USA

**Ediz Yesilkaya**

Turkey

**Junlin Yi**

China

**Panayiotis Yiallouros**

Cyprus

**Yee Ian Yik**

Malaysia

**Sze Lin Yoong**

Australia

**Christine Yoshinaga-Itano**

USA

**Kimberly Young**

USA

**Shi-Hui Yu**

USA

**Lourdes Yun**

USA

**Matthew Yung**

UK

**Kamran Yusuf**

Canada

**Minghao Zhong**

USA

**Ying Zhou**

USA

**Eyal Zimlichman**

Israel

**Elizabeta Zisovska**

Macedonia

**Stefano Zucchini**

Italy

**Katharine Zuckerman**

USA

**Warren Zuckerman**

USA

**John Zuniga**

USA

**Nicholas Zyromski**

USA

